# Supplementary material for: The interactome of intact mitochondria by cross-linking mass spectrometry provides evidence for coexisting respiratory supercomplexes
Source: Mol Cell Proteomics. 2017 Dec 8;17(2):216–32. doi: 10.1074/mcp.RA117.000470 (PMC5795388; doi:10.1074/mcp.RA117.000470)
Supplement: Supplemental Data [file supp_RA117.000470_133922_0_supp_23978_fzffwf.zip › spectra_annotation/mito_spectra_annotation/298-1-7-1-10-1.pdf]

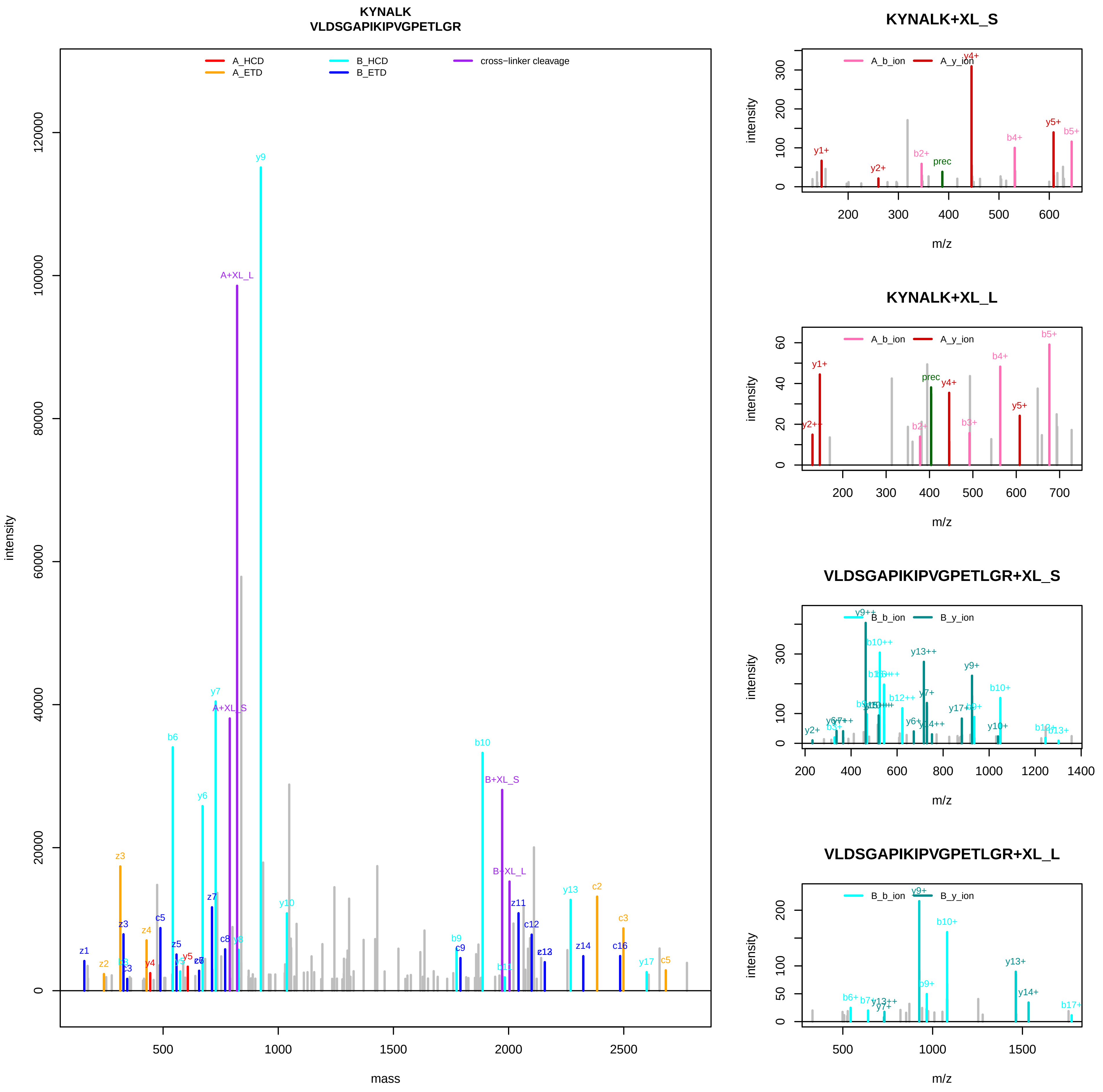
KYNALK
VLDSGAPIKIPVGPETLGR
A_HCD
A_ETD
B_HCD
B_ETD
cross−linker cleavage
y9
A+XL_L
y7
A+XL_S
b6
y6
b10
B+XL_S
B+XL_L
z3
y10
z7
z11
c12
y13
c2
c3
c16
z14
c13
c5
y17
z4
z5
c5
c8
y8
b9
c9
b11
z1
z2
c3
y4
y5
intensity
mass
KYNALK+XL_S
A_b_ion
A_y_ion
y1+
y2+
b2+
prec
y4+
b4+
y5+
b5+
m/z
KYNALK+XL_L
y2++
b3+
VLDSGAPIKIPVGPETLGR+XL_S
B_b_ion
B_y_ion
y9++
b10++
y13++
y9+
b10+
y7+
y17++
y10+
y6+
y14++
y2+
b13+
VLDSGAPIKIPVGPETLGR+XL_L
y13+
y14+
b6+
b7+
y7+
b9+
b17+
